# Histomorphological and Immunohistochemical Reappraisal of Cutaneous Adnexal Tumours: A Hospital Based Study

**DOI:** 10.1155/2016/2173427

**Published:** 2016-02-29

**Authors:** Prakriti Shukla, Uroos Fatima, Anil K. Malaviya

**Affiliations:** ^1^Department of Pathology, Era's Lucknow Medical College and Hospital, Lucknow, Uttar Pradesh, India; ^2^Bhopal Memorial Hospital and Research Center, Raisen Road, Bhopal, Madhya Pradesh 462038, India

## Abstract

*Background*. Diagnosing adnexal tumours of the skin is a challenge due to their wide variety, infrequent occurrence in practice, and confusing morphological picture.* Aims and Objectives*. The present study aims to observe the spectrum of adnexal tumours at our institute and to evaluate them based on histomorphological, histochemical, and immunohistochemical methods either alone or in combination for proper identification and classification.* Materials and Methods*. A partly retrospective and partly prospective study was conducted on adnexal skin tumours over a period of 6 years. Relevant clinical profile was recorded. Histopathological examination was carried out and special stains were applied as and when required. Immunohistochemistry was performed where diagnosis with routine stains was not possible.* Results*. A total of 150 skin tumour biopsies were received. There were 87 keratotic tumours, 39 adnexal tumours, and 24 melanocytic tumours. Amongst the adnexal tumours, 51.3% eccrine, 30.8% follicular, and 17.9% sebaceous tumours were seen. In five cases, histological diagnosis was troublesome where immunohistochemistry helped in making final diagnosis.* Limitations*. The sample size is small.* Conclusion*. Histomorphology is confirmatory in majority of the adnexal tumours but few rare lesions that mimic internal malignancy require a panel of immunomarkers to rule out other possible differentials.

## 1. Introduction

Adnexal tumours are a large and diverse group of neoplasms, classified morphologically into sweat gland tumours, hair follicle tumour, and sebaceous gland tumours. Diagnosis is a challenge due to wide variety of tumours, infrequent occurrence in practice, and confusing morphological features. Difficulty in classification of most of the adnexal tumours on the basis of clinical characteristics alone has earned them the name of “troublesome tumours” [1]. However, considering them benign without proper identification might result in serious consequences such as their transformation into metastatic form. This may be attributed to the acquisition of additional genetic events or to immunosuppression due to an underlying neoplastic disease [2].

Though most adnexal tumours are benign in nature, yet it is important to diagnose them accurately since many of them are genetically predetermined and may arise in the form of multiple potentially disfiguring lesions or may represent sites of development of more aggressive tumours at a later date. They may themselves be locally aggressive or capable of metastases and may be misdiagnosed as metastatic tumours to the skin [3].

Emergence of newer diagnostic techniques like immunohistochemistry in combination with histopathology has helped in differentiating and identifying these epidermal tumours precisely. Immunostains have made it easier to diagnose these tumours very accurately and helped dermatologists in predicting the prognosis correctly [4–6].

## 2. Material and Methods

A partly retrospective and partly prospective study was conducted on all the adnexal tumours of skin received in the Department of Pathology, at our institute over a period of 6 years from January 2006 to December 2011. This included the archival material of histologically diagnosed cases registered in the department as well as new cases received during the period of study from January 2011 to December 2011. The duration of the study was 5 years in the retrospective group and 1 year in the prospective group.

### 2.1. Inclusion Criteria

All clinically diagnosed cases of cutaneous adnexal tumours were included in the study.

### 2.2. Exclusion Criteria

Exclusion criteria are as follows:

1. Cases with inadequate specimen for pathological evaluation.
2. Cases of prospective study unwilling to go for biopsy wherever required.

Relevant information regarding age, sex, site, size, signs and symptoms, occupation, and family history was recorded. Histopathological examination was carried out in all cases. Special stains like Periodic Acid-Schiff (PAS), Alcian blue, and Van Gieson were applied as and when required to support the diagnosis. Immunohistochemistry was performed where pathogenesis was not clear and diagnosis with routine stains and histochemical stains was not possible. Sections were immunostained by streptavidin-biotin technique with primary antibodies to S-100, Vimentin, CK5/6, pan-cytokeratin (AE1/AE3), CD10, CD34, CK7, p63, epithelial membrane antigen (EMA), and carcinoembryonic antigen (CEA). WHO classification with its modification was the basis of our evaluation for adnexal tumours.

### 2.3. Statistical Tools Employed

The data so collected was subjected to statistical analysis using Statistical Package for Social Sciences version 15.0. The data has been presented in numbers and percentages and in mean ± SD (standard deviation).

## 3. Results

A total of 150 skin tumour biopsies were received in the histopathology department from years 2006 to 2011. Out of 150 cases, retrospective cases were 121 (80.7%) and prospective cases were 29 (19.3%) in number. There were 87 (58%) keratotic tumours, 39 (26%) adnexal tumours, and 24 (16%) melanocytic tumours.

Of these 39 adnexal tumours, 26 were benign (66.7%) and the remaining 13 (33.3%) were malignant in nature. Out of them, 20 (51.3%) were eccrine, 12 (30.8%) were follicular, and 7 (17.9%) were sebaceous tumours. The commonest age group was between 21 and 40 years (41%) followed by 41 and 60 years (35.9%) (Table 1). Males outnumbered the females with M : F ratio of 1.2 : 1. The commonest site was head, neck, and face (61.5%), followed by extremities (23.1%) and trunk (15.4%) (Table 2). Amongst them, 70% of the patients were of low socioeconomic status and were farmers, labourers, or vendors by profession (Table 3). Out of these thirty-nine cases, 86.84% of the patients presented with nodule, 7.90% presented with papule, and 5.26% showed an eczematous lesion. Majority of these cases had a tumour size ranging between 1.1 cm and 2 cm (Table 4).

The nature of different types of malignant or benign tumours was in accordance with their respective classification. Among the eccrine and apocrine types, nodular hidradenoma (4), eccrine spiradenoma (3), chondroid syringoma (2), eccrine poroma (2), Paget's disease (2), syringocystadenoma papilliferum (2), hidradenoma papilliferum (1), syringoma (1), and three cases of eccrine carcinoma were seen in the present study. Amongst the follicular types, pilomatricoma (4), proliferating trichilemmal tumour (4), trichoepithelioma (2), trichofolliculoma (1), and trichilemmoma (1) were observed. Out of 7 sebaceous tumours, sebaceous carcinomas (4), sebaceoma (1), and sebaceous adenomas (2) were noted in the present study (Table 5).

There were five cases where histological diagnosis was troublesome. These cases were reevaluated in the light of special stains and immunohistochemistry for the final diagnosis.

To differentiate sweat gland carcinoma from cutaneous metastasis, we applied p63, CK5/6, and CEA. The luminal cells were positive for CEA and CK5/6 while myoepithelial cells expressed strong immunoreactivity for p63 suggesting its origin from eccrine sweat gland. Based on morphology and immunoprofile, it was diagnosed as digital papillary adenocarcinoma.

The second case was of desmoplastic trichoepithelioma which was simulating basal cell carcinoma. Immunohistochemistry revealed CD10 positivity in the tumour cells and strong CD34 expression in the stromal cells. CK5/6 was also applied in this case.

The third case was diagnosed as trichilemmal carcinoma and it was differentiated from squamous cell carcinoma by the application of pan-cytokeratin (AE1/AE3), EMA, and p63. Immunohistochemistry showed tumour cells positivity for pan-cytokeratin (AE1/AE3), but epithelial membrane antigen (EMA) and p63 were negative.

Another rare case of chondroid syringoma was diagnosed based on histomorphological and immunohistochemical findings. The tumour cells displayed widespread and strong positivity for CEA and the outer layers of cells were positive for S-100 and Vimentin.

The last case was of hidradenoma papilliferum occurring in a male patient with extra genital site. In this case, the tumour typically expressed CK7, EMA, CEA, and GCDFP 15.

The salient histomorphological features of different eccrine (Table 6), follicular (Table 7), sebaceous (Table 8), and other troublesome tumours (Table 9) have been described.

## 4. Discussion

Cutaneous adnexal tumours were first recognised in the later part of nineteenth century. They are relatively uncommon and are thought to have a genetic basis. Diagnosis is essentially based on histopathological examination as clinical features are not very distinctive. They may sometimes display more than one line of histological differentiation resembling other wide variety of tumours. In such cases, immunohistochemistry and ultrastructural studies may aid in establishing the accurate diagnosis.

In the present study, only 39 adnexal tumours were seen over a period of 6 years among 23,000 pathology records. Other authors have reported a lower prevalence with only 112 cases reported over a 13-year survey of consecutive biopsies in Malaya [7]. In another study in Nigeria [8], 52 adnexal tumours were seen over a 16-year period, accounting for 0.9% of all cutaneous tumours. The true incidence of adnexal tumours is believed to be higher than the actually described in the literature because many of them either are asymptomatic so the patients do not report or are treated with destructive modalities without any prior biopsy [9].

Out of 39 adnexal tumours, 51.3% were eccrine and apocrine type, 30.8% were pilar type, and 17.9% were sebaceous type. Majority of the cases were seen in the age group of 21 to 60 years and the commonest site was head, neck, and face with male : female ratio of 1.2 : 1. Maximum cases were benign (66.7%) and 70% of the patients were farmers and labourers. Most of them presented as nodules (86.84%) of size above 1.2 cm (76.3%). Yaqoob et al. [10], in a study of adnexal tumours at Karachi, found that proportion of eccrine and apocrine tumours was maximum (51.9%) with mean age of 41.72 years and the commonest site was head and neck. There was no sex predilection in their study and 87.3% were benign. Jindal and Patel [11] found 96% of the benign adnexal neoplasms in their study and most of them were localized to head and neck region with 48% being sweat gland tumours and 40% were of pilosebaceous type. Majority of these tumours were less than 2 cm in their study. Thus, the clinical parameters in the present study are similar to others but the important part has been the increased incidence among people exposed to sun.

**Figure 1:**
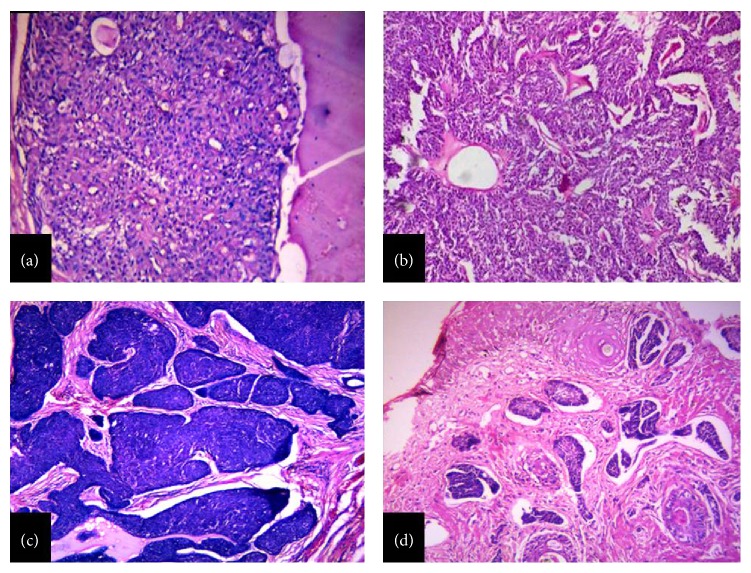
(a) Nodular hidradenoma, showing clear and polygonal cells with foci of eosinophilic material (H&E, ×100). (b) Hidradenoma papilliferum, showing scattered cystic and branching spaces with numerous papillary projections lined by double layer of epithelium (H&E, ×100). (c) Eccrine spiradenoma, showing well circumscribed aggregates of tumour cells in dermis (H&E, ×100). (d) Syringoma, showing numerous small ducts embedded in a fibrous stroma (H&E, ×100).

The nature of different types of malignant or benign tumours was in accordance with their respective classification. Amongst the eccrine and apocrine types, nodular hidradenoma was the commonest neoplasm. The age range was between 40 and 76 years and there was no sex predilection. All cases presented with solitary, circumscribed, solid and cystic, dermal, and lobulated neoplasm with sheet-like and papillary architecture. The tumour cells were eosinophilic with a regular oval nucleus and a small inconspicuous nucleolus. Clear cells were also present in abundance (Figure 1). Similar findings were observed by other authors [10, 11].

Hidradenoma papilliferum (Figure 1) is a cystic and papillary apocrine neoplasm which characteristically affects women above 30 years of age and occurs mainly in the anogenital region [3]. Minami et al. [12] reported it in a 52-year-old male in the eyelid. In the present study also, it was found in a 16-year-old male involving upper and lower eyelids of left eye. Owing to its uncommon clinical presentation, immunostaining was performed using CK7, CEA, EMA, and GCDFP 15. The result revealed positivity for the above markers.

Tumours like eccrine spiradenoma, eccrine poroma, syringocystadenoma papilliferum, and syringoma presented no difficulty in diagnosis (Figure 1).

**Figure 2:**
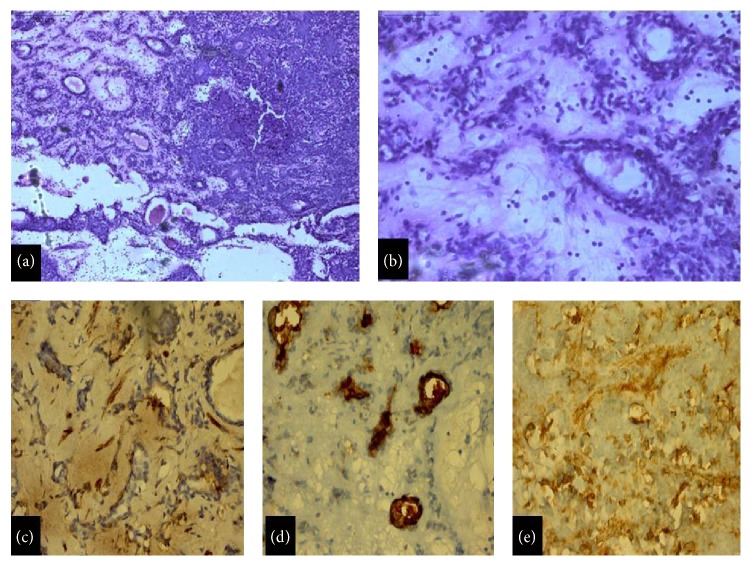
Chondroid syringoma (a) branching tubular epithelial cells in basophilic stroma (H&E, ×50). (b) Tubular lumina lined by cuboidal cells (luminal) and flat cells (periphery) (H&E, ×400). (c) Outer cell layer showing S-100 positivity (Immunoperoxidase, ×100). (d) Luminal cells showing CEA positivity (Immunoperoxidase, ×100). (e) Outer cell layer showing Vimentin positivity (Immunoperoxidase, ×100).

An interesting case of chondroid syringoma was seen in the present study which showed branching tubular epithelial cells in basophilic stroma (Figure 2). The tubular lumina were lined by cuboidal cells (luminal) and flat cells (periphery) in a mucoid stroma. Histomorphology alone was not convincing, so immunohistochemistry was done using CEA, Vimentin, and S-100. The luminal cells showed positivity for CEA while outer cells were Vimentin and S-100 positive. This finding helped in diagnosing chondroid syringoma.

In the present study, three cases of eccrine carcinoma had involvement of scalp and upper extremities: two cases were seen in females and one case was seen in a male. The patients were 22 years, 50 years, and 45 years old, respectively. Eccrine carcinoma usually involves individuals of middle to old age and predominantly affects females [13]. In the present study, findings were in accordance with the other authors' findings.

**Figure 3:**
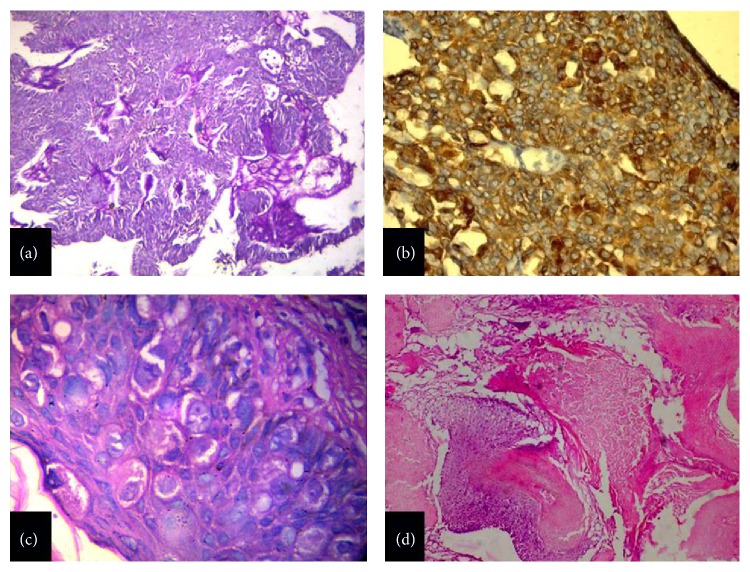
(a) Sweat gland tumour, showing prominent papillary epithelial projections (H&E, ×50). (b) Sweat gland tumour, showing CK5/6 positivity in the tumour cells (Immunoperoxidase, ×100). (c) Paget's disease, showing large rounded Paget cells with ample pale staining cytoplasm (H&E, ×400). (d) Pilomatricoma, showing rounded basophilic cells and shadow cells with loss of nuclei and distinct border (H&E, ×50).

Among the eccrine carcinomas, two cases were diagnosed as malignant mixed tumour and mucinous tumour, respectively, while in the third case histomorphology was troublesome as it was mimicking a cutaneous metastasis. To evaluate its histogenesis, we performed immunohistochemistry using markers like p63, CEA, and CK5/6 [14–16]. The cells in the excretory coil expressed CEA and CK5/6 supporting the diagnosis of a sweat gland tumour. It was diagnosed as digital papillary tumour with papillary projections lined by columnar epithelium within cystic spaces in the dermis (Figure 3). The cysts showed decapitation secretion. Mitosis and necrosis were abundant.

Paget's disease of the nipple is an unusual epidermal presentation of underlying breast cancer (Figure 3). It presents as an eczematous change or erythematous ulceration but may also be an incidental histological finding in a mastectomy specimen [17, 18]. In the present study also, an eczematous lesion was seen.

Among the follicular tumours, pilomatricoma is a relatively common benign cutaneous adnexal neoplasm with differentiation towards the matrix and inner sheath of a normal hair follicle as well as hair cortex. In the present study too, it was found to be the most common among adnexal neoplasms with follicular origin. The tumour occurs in all age groups [19]. In the present study, age of patients varied from 27 to 98 years, thus showing prevalence across all the age groups. In the present study, male to female ratio was 1 : 1 with both the genders having 2 cases each. Histological findings of shadow cells, basaloid cells, and calcification were characteristic (Figure 3).

**Figure 4:**
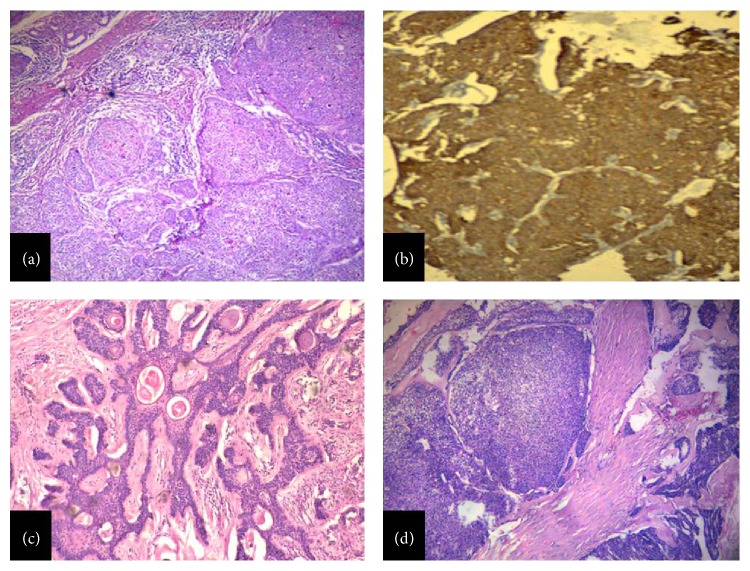
(a) Trichilemmal carcinoma, invasive tumour with solid pattern showing clear cells (H&E, ×50). (b) Trichilemmal carcinoma, showing pankeratin (AE1/3) positivity in the tumour islands (Immunoperoxidase, ×50). (c) Trichofolliculoma, showing keratin cysts lined by squamous epithelium emanating from which numerous secondary hair follicles are seen (H&E, ×50). (d) Sebaceous carcinoma, showing numerous irregular lobules with infiltrative growth in the dermis (H&E, ×50).

Malignant proliferating trichilemmal tumours (MPTT) are rare neoplasm arising from outer root sheath of hair follicle and have been shown to occur in late middle and elderly age group predominantly among females [20, 21]. In the present study also, none of the cases were below 40 years of age and had a female preponderance (3 : 1) and all had involvement of head. One case resembled squamous cell carcinoma which was then reevaluated by performing IHC with markers like pan-cytokeratin (AE1/AE3), EMA, and p63. The results of the above markers showed differentiation towards follicular epithelial origin (Figure 4).

Trichoepithelioma, trichilemmoma, and trichofolliculoma were seen on head and neck with male preponderance and the findings were characteristic. In trichoepithelioma, horn cysts with variable basaloid epithelial formations were seen while, in trichofolliculoma, keratin filled cysts with immature follicles were seen (Figure 4). In trichilemmoma, verrucous hyperplasia with lobular formations having clear cells was the diagnostic feature. There was one case of desmoplastic trichoepithelioma which needed differentiation from basal cell carcinoma. Here, IHC was done using CD34, CD10, and CK5/6. In our case, the tumour cells showed negativity with CD34 but stromal cells showed diffuse positivity. CD10 and CK5/6 positivity was seen in the tumour cells. CK 5/6 positivity is seen in both DT and BCC. It was done only as an adjunct to assess the origin of basaloid cells seen in DT [22–24].

Among the sebaceous tumours, sebaceous carcinoma (Figure 4) was the commonest. It usually arises in adults, with an average age of 62 years and a female predominance, by a factor of roughly 2 : 1. Tumours of the eyelids are preferentially seen in Asian patients [25]. In the present study, all the 4 cases had involvement of eyelids and were aged between 52 and 74 years with an average age of 60 years but a male predominance was seen. A male predominance of sebaceous carcinoma has been reported in extra ocular regions by Lazar et al. [26]. Thus, in the present study, the distinguishing finding in cases of sebaceous carcinoma was a male predominance.

All the benign cases, that is, sebaceoma and sebaceous adenoma, showed preponderance of head, neck, and face region and had a male preponderance. However, it is difficult to comment on these as solitary cases cannot be generalized.

In the present study, we have avoided generalized inference based on solitary cases as even solitary cases have some specific presentation.

## 5. Conclusion

Histomorphology was confirmatory in majority of the tumours but not much convincing in few uncommon lesions of adnexal origin on account of their unusual clinical presentation and also because of their overlapping features with internal malignancy. The tumours causing diagnostic difficulty by conventional histology were subjected to immunohistochemistry on sections from formalin fixed paraffin embedded material.

There were five such adnexal tumours in the present study where histomorphology alone was not sufficient to clinch a proper diagnosis. There we applied special stains and immunohistochemistry to reach a diagnosis. It is concluded that use of immunostaining of cellular antigens like Vimentin, S100, EMA, CEA, GCDFP 15, CD10, p63, CD34, pan-cytokeratin (AE1/AE3), CK5, CK6, and CK7 is helpful in diagnosing such tumours. It is essential to perform a panel of immunomarkers in difficult cases to rule out other possible differentials.

## Figures and Tables

**Table 1:** Age-wise distribution of adnexal tumours.

SN	Age (years)	Adnexal tumours (*n* = 39)	
		Number	Percentage
1	≤20	2	5.1
2	21–40	16	41.0
3	41–60	14	35.9
4	61–80	5	12.8
5	81–100	2	5.1

**Table 2:** Site/location of adnexal tumours.

SN	Site	Adnexal (*n* = 39)	
		Number	Percentage
1	Head, neck, and face	24	61.5
2	Extremities	9	23.1
3	Trunk	6	15.4

**Table 3:** Distribution of adnexal tumours according to the occupation.

SN	Occupation	Adnexal (*n* = 10)	
		Number	Percentage
1	Housewife	1	10.0
2	Farmer/labourer/vendor	7	70.0
3	Businessperson	0	0.0
4	Policeman/teacher	1	10.0
5	Student	1	10.0

**Table 4:** Distribution of adnexal tumours according to type and size of skin lesion (*n* = 38).

SN	Type of skin lesion	Number	%	Size of skin lesion	Number	%
1	Eczematous	2	5.26	≤1 cm	4	10.5
2	Nodule	33	86.84	1.1–2 cm	29	76.3
3	Papule	3	7.90	2.1–3 cm	3	7.9
4	Ulcer	0	0	3.1–4 cm	2	5.3

**Table 5:** Clinical profile of different adnexal tumours.

SN	Final diagnosis	Number of cases	Mean age ± SD (range)	M : F	Location
Eccrine and apocrine (*n* = 20)					
1	Eccrine spiradenoma	3	40.00 ± 13.23 (25–50)	1 : 2	Extremities (*n* = 2), trunk (*n* = 1)
2	Nodular hidradenoma	4	55.00 ± 30.56 (40–76)	2 : 2	Head, neck/face (*n* = 1), extremities (*n* = 2), and trunk (*n* = 1)
3	Chondroid syringoma	2	46.50 ± 14.85 (36–57)	2 : 0	Head, neck/face (*n* = 1), extremities (*n* = 1)
4	Eccrine carcinoma	3	39.00 ± 14.93 (22–50)	1 : 2	Head, neck/face (*n* = 1), extremities (*n* = 2)
5	Eccrine poroma	2	27.50 ± 3.54 (25–30)	1 : 1	Head, neck/face (*n* = 1), trunk (*n* = 1)
6	Paget's disease of nipple	2	61.50 ± 34.65 (37–86)	0 : 2	Trunk (*n* = 2)
7	Syringocystadenoma papilliferum	2	24.00 ± 8.49 (18–30)	1 : 1	Head, neck/face (*n* = 2)
8	Hidradenoma papilliferum	1	16.00	1 : 0	Head, neck/face (*n* = 1)
9	Syringoma	1	22.00	0 : 1	Head, neck/face (*n* = 1)

Follicular (*n* = 12)					
1	Pilomatricoma	4	55.50 ± 30.56 (27–98)	2 : 2	Head, neck/face (*n* = 2), extremities (*n* = 1), and trunk (*n* = 1)
2	Proliferating trichilemmal tumour	4	51.67 ± 10.41 (40–60)	1 : 3	Head, neck/face (*n* = 4)
3	Trichoepithelioma/desmoplastic trichoepithelioma	2	60.00 ± 21.21 (45–75)	2 : 0	Head, neck/face (*n* = 2)
4	Trichofolliculoma	1	32.00	1 : 0	Head, neck/face (*n* = 1)
5	Trichilemmoma	1	38.00	1 : 0	Head, neck/face (*n* = 1)

Sebaceous (*n* = 7)					
1	Sebaceous carcinoma	4	60.25 ± 10.21 (52–74)	3 : 1	Head, neck/face (*n* = 4)
2	Sebaceoma	1	62.00	1 : 0	Upper extremity (*n* = 1)
3	Sebaceous adenoma	2	(26–24)	1 : 1	Head, neck/face (*n* = 2)

**Table 6:** Salient histopathological features of eccrine and apocrine tumours.

Malignant mixed tumour	(i) Composed of epithelial and chondromyxoid mesenchymal components. (ii) Epithelial tumour aggregates seen as confluent cords and nests with interspersed zones of tubule formation. (iii) Numerous mitotic figures and zones of necrosis seen.

Mucinous carcinoma	(i) The tumour cells in numerous compartment divided by strands of fibrous tissue. (ii) In each compartment, abundant amount of pale staining mucin seen around the nests of anaplastic epithelial cells. (iii) Some showing tubular lumen and focal duct formations.

Paget disease of breast	(i) Epidermis with scattered Paget cells seen which were large, rounded cells devoid of intercellular bridges containing a large nucleus with ample cytoplasm. (ii) The dermis showing moderately severe chronic inflammatory reactions.

Syringoma	(i) Numerous small ducts embedded in a fibrous stroma. (ii) Few ducts that had small, comma-like tails of epithelial cells giving appearance of tadpoles. (iii) Solid strands of basophilic epithelial cells independent of ducts also seen.

Poroma	(i) Tumour consisting of broad, anastomosing bands from epidermis. (ii) Tumour cells which are small, cuboidal, and basophilic. (iii) Cystic spaces lined by eosinophilic PAS positive, diastase resistant cuticle.

Hidradenoma	(i) Multiple lobules of lesional cells. (ii) Tumour comprised of clear and polygonal cells. (iii) Foci of eosinophilic material and small preductal lumina seen. (iv) Focal cystic changes.

Syringocystadenoma papilliferum	(i) Tumour comprised of numerous papillary projections. (ii) Papillary projections lined by two rows of cells-luminal row-columnar cells with active decapitation secretion, outer row-cuboidal cells. (iii) The secretions seen as cystic invaginations extending into the dermis.

Eccrine spiradenoma	(i) Well circumscribed aggregates of tumour cells seen in dermis. (ii) Two types of cells, at the periphery, cells with small dark nuclei, and in the center, large cells with pale nuclei. (iii) Eosinophilic material in the lumina.

**Table 7:** Salient histopathological features of follicular tumours.

Proliferating trichilemmal tumour	(i) Tumour lobules of squamous epithelium (ii) Epithelium in centre abruptly changed into amorphous keratin (iii) Epidermoid keratinization resembling squamous eddies with nuclear atypia and individual cell keratinisation

Pilomatricoma	(i) Tumour made of epithelial islands in a cellular stroma, located in dermis containing two types of cells (ii) Basophilic cells round to elongated (iii) Shadow cells that had distinct border with loss of nucleus and calcification seen

Trichilemmoma	(i) Verrucous hyperplasia with lobular formations extending into the dermis (ii) Many cells demonstrating clear cytoplasm (PAS positive)

Trichofolliculoma	(i) Keratin filled cysts lined by squamous epithelium (ii) Numerous secondary (immature) hair follicles emanating from the cyst wall

Trichoepithelioma	(i) Superficial dermal lesion with prominent stroma (ii) Horn cyst of variable sizes (iii) Basaloid epithelial formations (iv) No retraction artifact

**Table 8:** Salient histopathological features of sebaceous tumours.

Sebaceous carcinoma	(i) Irregular epithelial lobules with infiltrative growth in dermis (ii) Lesional cells, marked cytological atypia, and focal sebaceous differentiation

Sebaceous adenoma	(i) Tumour composed of sebaceous lobules of varying size and shape (ii) Two types of cells: basaloid cells and sebaceous cells (iii) Predominant component, sebaceous cells

Sebaceoma	Majority of the lesion showing undifferentiated basaloid cells with islands of sebaceous cells and occasional ducts

**Table 9:** Salient histopathological feature of troublesome tumours.

Sweat gland tumour (digital papillary tumour)	(i) Multinodular epithelial aggregates with cystic spaces in the dermis. (ii) Papillary epithelial projections within cystic spaces associated with fibrovascular cores in some areas. (iii) The epithelium made of low columnar cells and cysts filled with eosinophilic material. (iv) Mitoses and necrosis seen.

Hidradenoma papilliferum	(i) Dermal nodule with scattered cystic and branching spaces. (ii) Numerous papillary projections lined by single layer of high cylindric cells with a basal layer. (iii) Active decapitation secretions.

Chondroid syringoma	(i) Nodular tumour. (ii) Branching tubular epithelial cells in basophilic stroma. (iii) Tubular lumina lined by cuboidal cells (luminal) and flat cells (periphery). (iv) Mucoid stroma.

Trichilemmal carcinoma	(i) Invasive tumour with solid and trabecular pattern seen. (ii) Cytologically atypical clear cells with foci of pilar type keratinization.

Desmoplastic trichoepithelioma	(i) Narrow strands of tumour cells (ii) Numerous horn cysts (iii) Desmoplastic stroma (iv) Areas of calcification
